# From lipid overload to autophagy collapse: how lipid dysregulation drives chronic inflammation and metabolic disease

**DOI:** 10.1007/s00011-026-02283-w

**Published:** 2026-06-11

**Authors:** Mykyta Fomin, Konstantin Zapf, Elisabeth Schieffer, Johannes Freitag, Gert Bange, Bernhard Schieffer

**Affiliations:** 1 Marburg University, Marburg, Germany; 2 Department of Medicine, Marburg University, Marburg, Germany; 3 Department of Cardiology, Angiology and Critical Care Medicine, University Hospital Marburg (UKGM), Baldingerstrasse 1, 35047 Marburg, Germany; 4 Center for Postinfectious Disease (CEPISM), Marburg University, Marburg, Germany; 5 Departments of Chemistry and Biology, Center for Synthetic Microbiology (Synmikro), Marburg University, Karl-von-Frisch Strasse 14., 35043 Marburg, Germany

**Keywords:** Autophagy, Cholesterol fractions, Diseases, Inflammation, Lipid metabolism, Pathway analysis

## Abstract

**Introduction:**

Autophagy is a central homeostatic mechanism that preserves intracellular quality control by clearing damaged organelles, aggregated proteins, and excess lipids. Increasing evidence indicates that the lipid-autophagy axis is a critical determinant of chronic inflammatory and metabolic disease. Cholesterol-rich and oxidatively modified lipoproteins, including very-low-density lipoprotein (VLDL), low-density lipoprotein (LDL), oxidized LDL, and lipoprotein(a), can impose lysosomal stress, disturb autophagosome maturation, and amplify oxidative and inflammatory signaling, whereas high-density lipoprotein-mediated cholesterol efflux supports cellular lipid clearance and autophagic competence. When chronic lipid overload exceeds lysosomal and autophagic capacity, cells transition from adaptive lipophagy to impaired autophagic flux, leading to lipid-droplet accumulation, mitochondrial dysfunction, inflammasome activation, and sustained cytokine production. This review synthesizes mechanistic insights linking lipid dysregulation and autophagy failure across atherosclerosis, metabolic dysfunction-associated steatotic liver disease/metabolic dysfunction-associated steatohepatitis (MASLD/MASH), and neurocognitive disorders. We further discuss how defective autophagy impairs efferocytosis, phagosome maturation, and inflammasome restraint, thereby contributing to unresolved inflammation and inflammatory cell-death signaling. Translationally, we outline therapeutic strategies that combine metabolic unloading, lipid-lowering interventions, autophagy-lysosome modulation, and flux-based biomarker approaches.

**Conclusion:**

Lipid-induced autophagic flux failure provides a unifying framework for understanding how metabolic stress evolves into chronic inflammation and organ dysfunction and identifies actionable targets for precision therapeutic intervention.

## Introduction

Autophagy is a fundamental protective mechanism that enables eukaryotic organisms to maintain overall vitality and supports tissue renewal throughout life by facilitating the controlled removal of dysfunctional or damaged material [1, 2]. Although discovered relatively late (in the 1990s) in *Saccharomyces cerevisiae* screenings due to the subtle phenotypes associated with the absence of autophagy, today it is clear that autophagy is a fundamental process in eukaryotic organisms [3, 4]. Understanding the molecular-mechanistic details of the process is emerging but remains relatively incomplete [2].

Various studies have demonstrated that dysfunction of autophagic processes contributes to a broad spectrum of diseases, including cardiovascular, metabolic, and neurodegenerative disorders [5]. Atherosclerosis exemplifies this link: immune dysregulation and perturbed lipid metabolism drive plaque development, while defective autophagy accelerates foam-cell formation, inflammation, and plaque instability. Similarly, metabolic dysfunction-associated steatotic liver disease (MASLD; formerly NAFLD) is closely linked to impaired hepatic autophagy, reduced lipid-droplet turnover, defective free fatty acid mobilization, and progressive metabolic inflammation [6–8].

Given its central role in energy homeostasis, signal transduction, and membrane biogenesis, lipid metabolism constitutes a critical dimension of autophagy regulation. Cholesterol-transporting lipoproteins and their apolipoproteins are especially important because they coordinate systemic lipid flow and help sustain cellular metabolic balance. Cholesterol, a crucial structural component of eukaryotic membranes, has been linked to the regulation of autophagy through various mechanisms, including the modulation of lysosomal function, membrane fluidity, and ROS-dependent signaling [9–11]. Although the exact nature of these interactions is still under debate, cholesterol depletion often correlates with increased expression of autophagy-related genes, possibly through oxidative-stress pathways [11].

Lipoproteins such as VLDL, LDL, oxLDL, and HDL differ significantly in density, composition, and biological roles, with profound consequences for autophagic regulation [12]. Cholesterol-rich lipoproteins (CRLs) promote inflammatory signaling and disrupt autophagic homeostasis [13–15], whereas HDL facilitates cholesterol efflux and supports autophagic activity, thereby reducing cellular stress [16, 17]. Beyond classical cardiometabolic disorders, persistent inflammatory states may further amplify autophagy dysfunction by sustaining mitochondrial stress, lysosomal overload, and inflammasome activation [18]. This review first outlines the molecular interface between lipid metabolism, lipophagy, and autophagic flux. It then discusses how lipid overload disrupts lysosomal and mitochondrial quality control. Finally, it examines how these mechanisms promote chronic inflammation in cardiovascular disease, metabolic liver disease, and neurocognitive decline.

## Lipoprotein metabolism and cellular lipid homeostasis

To understand how lipid dysregulation affects autophagy, it is first necessary to define the major circulating lipoprotein fractions and their roles in systemic lipid transport. Generally, lipids circulate as complex particles that can undergo various structural and biochemical modifications, often as lipoproteins. These particles are heterogeneous complexes composed of lipids and proteins and serve as transport vehicles for the absorption, utilization, and distribution of dietary and endogenous fats, including cholesterol [19].

Advances in lipoprotein isolation techniques over the past decades have led to two major insights: first, it has become evident that lipoproteins can be separated into distinct fractions, which primarily differ in their cholesterol and triglyceride content. Furthermore, determination of the specific lipoprotein phenotype is of great clinical importance, as therapeutic strategies may vary substantially depending on the phenotype. In recent decades, improved analytical methods, such as ultracentrifugation, NMR profiling, and mass spectrometry-based lipidomics, have revealed several clinically relevant lipoprotein phenotypes. The most widely recognized are lipoprotein phenotypes A and B: phenotype A, defined by larger, more buoyant LDL particles, and phenotype B, characterized by small, dense LDL particles with a higher atherogenic risk. Additional important phenotypes include elevated Lipoprotein(a), triglyceride-rich VLDL remnants, and dysfunctional HDL subclasses. These advances have allowed a more precise classification of lipoprotein patterns and have directly informed phenotype-specific treatment approaches [19].

Seven major classes of lipoproteins have been identified based on their particle size, lipid composition, and associated apolipoproteins: chylomicrons, chylomicron remnants, very-low-density lipoproteins (VLDL), intermediate-density lipoproteins (IDL), low-density lipoproteins (LDL), high-density lipoproteins (HDL), and an LDL-like particle containing apolipoprotein (a) [20]. Recent studies have demonstrated clear correlations between lipoprotein fractions and disease risk. Elevated levels of LDL cholesterol and triglycerides are consistently associated with an increased risk of cardiovascular disease (CVD) [21, 22]. In contrast, higher HDL cholesterol concentrations have traditionally been associated with lower CVD risk, although HDL functionality and cholesterol efflux capacity may be more informative than HDL cholesterol levels alone [21]. Another important aspect involves the influence of sex and age on lipoprotein concentrations; however, these parameters are not the focus of the present review, which dissects the general biochemical mechanism and clinical data related to lipid metabolism and autophagy [20]. Table 1 summarizes the composition and essential functions of the major lipoprotein fractions [23]. These will be discussed in detail below:

**Table 1:** Summary of the major lipoprotein fractions and their composition and essential functions

Lipoprotein fraction	Apolipoproteins	Density [g/ml]	Lipid	Origin	Physiologic function
Chylomicrons	ApoB-48, ApoC-II, ApoE	< 0.95	Triglycerides (dietary)	Intestine	Transport of dietary triglycerides and cholesterol from the intestine to peripheral tissues (muscle, adipose)
VLDL	ApoB-100, ApoC, ApoE	0.95–1.006	Triglycerides (endogenous)	Liver	Export of hepatic triglycerides to peripheral tissues; precursor of IDL and LDL
IDL	ApoB-100, ApoE	1.006–1.019	Triglycerides + Cholesteryl esters	Catabolic product of VLDL	Transitional particle between VLDL and LDL; partly cleared by hepatic ApoE receptors
LDL	ApoB-100	1.019–1.063	Cholesteryl esters	VLDL/IDL catabolism	Major carrier of plasma cholesterol to peripheral tissues via LDL receptor-mediated endocytosis
HDL	ApoA-I, ApoA-II	1.063–1.21	Phospholipids, Cholesteryl esters	Liver and intestine	Mediates reverse cholesterol transport; removes excess cholesterol from cells and delivers it to the liver
Lp(a)	ApoB-100 + Apo(a)	1.05–1.10	Cholesteryl esters	Liver	LDL-like particle containing Apo(a), which resembles plasminogen, may modulate fibrinolysis and vascular repair

**VLDL (Very-Low-Density Lipoprotein)** is a triglyceride-rich particle secreted by hepatocytes, composed of approximately 90% lipids and 10% protein, with apoB-100 serving as its structural core [23]. VLDL particles deliver endogenous triglycerides to peripheral tissues and can also promote pro-inflammatory signaling, particularly through apoC-III, thereby contributing to metabolic inflammation and endothelial dysfunction [23]. In addition, VLDL serves as the precursor of low-density lipoprotein (LDL), forming intermediate-density lipoproteins (IDL) during its catabolism [23].

**LDL (Low-Density Lipoprotein)**, the primary cholesterol carrier in plasma, arises from VLDL catabolism and is central to atherogenesis. LDL delivers cholesterol to peripheral cells via LDL receptors. Excess LDL can be internalized by macrophages, contributing to foam cell formation. After subendothelial retention, LDL can undergo oxidative modification, generating oxLDL, a highly pro-inflammatory and lipotoxic particle. Uptake of oxLDL promotes intracellular lipid accumulation, which may impair autophagic activity [23–25].

**Lipoprotein(a) (Lp(a))** is a structurally distinct lipoprotein, composed of an LDL-like particle covalently linked to apolipoprotein(a) (Apo(a)), a large glycoprotein homologous to plasminogen [26, 27]. Elevated Lp(a) levels are an established independent risk factor for atherosclerosis and aortic valve stenosis, contributing to disease through both prothrombotic effects (by competing with plasminogen) and pro-inflammatory mechanisms, primarily mediated by oxidized phospholipids (oxPLs) carried on the Apo(a) tail [26].

At the cellular level, lipid homeostasis is a tightly coordinated process that balances lipid uptake, storage, utilization, and efflux, thereby maintaining membrane integrity, energy homeostasis, and signaling fidelity. Under physiological conditions, excess lipids are stored in cytoplasmic lipid droplets, which serve as dynamic reservoirs mobilized during increased metabolic demand [28, 29]. When lipid influx exceeds cellular storage and catabolic capacity—such as during hyperlipidemia, lipotoxic stress, or impaired lipoprotein clearance—intracellular lipid accumulation becomes cytotoxic and perturbs organelle function [7, 29]. To mitigate these effects, cells engage autophagy-related mechanisms, particularly lipophagy, a selective form of autophagy that targets lipid droplets for lysosomal degradation [29]. Through lipophagy, stored neutral lipids are hydrolyzed within autolysosomes, the major cellular organelles for catabolism of lipid-containing compounds. This process releases free fatty acids, which can subsequently be oxidized in mitochondria or peroxisomes. Excessive lipid load can impair autophagic flux by disrupting lysosomal membrane integrity, altering membrane lipid composition, or modulating key regulators such as mechanistic target of rapamycin (mTOR) and AMP-activated protein kinase (AMPK) [7, 28, 30]. The interaction between lipid homeostasis and autophagy constitutes a crucial metabolic checkpoint that determines whether lipids are stored, mobilized, or degraded [29]. Dysregulation of these pathways contributes to metabolic and inflammatory disease processes, including atherosclerosis, foam-cell formation, and MASLD [7, 31]. Understanding the interfaces between lipoprotein trafficking and autophagic control is therefore essential for elucidating the cellular strategies that maintain lipid and energy homeostasis under stress conditions.

**HDL (High-Density Lipoprotein)** mediates reverse cholesterol transport by removing excess cholesterol from peripheral cells and delivering it back to the liver. This process can be enhanced by lipophagy, as macrophages in atherosclerotic plaques can mobilize cholesterol for HDL-mediated efflux. HDL is primarily synthesized in the liver and intestine and is rich in phospholipids and free cholesterol. Two key enzymes regulate HDL metabolism: lecithin-cholesterol acyltransferase (LCAT), which esterifies cholesterol to generate mature spherical HDL particles, and cholesteryl ester transfer protein (CETP), which mediates the exchange of cholesteryl esters and triglycerides between HDL, VLDL, and LDL [23] .

## Autophagy: core mechanisms and functional relevance

Autophagy comprises lysosome-dependent degradation and recycling pathways that preserve intracellular homeostasis by controlling the quantity and quality of proteins, lipids, and organelles. Although active at basal levels in most cells, autophagy is robustly upregulated under metabolic and environmental stressors, including nutrient deprivation, to generate essential substrates and energy [7, 32]. Beyond its classical catabolic function, autophagy critically shapes immune homeostasis by modulating antigen presentation, inflammasome activation, cytokine release, efferocytosis, and immune cell polarization. Increasing evidence indicates that impaired autophagy can act as an active pathogenic driver in chronic inflammatory diseases, rather than merely representing a secondary consequence of cellular stress [32–34]. When dysregulated, autophagy fails to eliminate damaged organelles and protein aggregates, leading to mitochondrial dysfunction, oxidative stress, sustained inflammasome activity, and persistent cytokine production. These mechanisms provide an important conceptual basis for understanding how defective autophagy can promote inflammatory cell-death signaling, including NLRP3 inflammasome activation, IL-1β maturation, gasdermin D cleavage, and pyroptosis [18, 35]. Autophagy pathways are categorized according to their route to the lysosome into macroautophagy, microautophagy, and chaperone-mediated autophagy (CMA). Specialized variants of these pathways selectively target defined substrates, including protein aggregates (aggrephagy), mitochondria (mitophagy), or lipid droplets (lipophagy) [7].

### Macroautophagy

Macroautophagy is a highly conserved lysosomal degradation pathway that involves the formation of double-membrane vesicles, termed autophagosomes, which sequester cytosolic cargo and subsequently fuse with lysosomes for degradation [7, 36]. The process can be amplified by diverse stimuli such as nutrient withdrawal, radiotherapy, or pharmacological treatments [7]. It begins with the formation of a phagophore, which elongates and closes to generate an autophagosome. Upon fusion with lysosomes, the autolysosome exposes its cargo to lysosomal hydrolases [7, 37]. Initiation requires the autophagy-activating serine/threonine-protein kinase complex (ULK1), which integrates signals from the energy sensors mTORC1 and AMPK [7, 38]. Macroautophagy can be nonselective (random sequestration of cytoplasm) or selective (degradation of specific cargoes such as aggregates, mitochondria, peroxisomes, or lipid droplets) [39, 40]. Selectivity is mediated by autophagy receptors that link cargo to the phagophore—either ubiquitin-dependent or ubiquitin-independent [39, 40]. Most receptors bind via LC3-interacting regions (LIRs) to microtubule-associated protein 1A/1B-light chain 3 (LC3) proteins on the inner phagophore membrane [41], thereby tethering cargo to the nascent vesicle. LC3 belongs to the autophagy-related protein 8 (Atg8) family of ubiquitin-like modifiers that are covalently conjugated to phospholipids in the autophagosomal membrane, functioning as an “ubiquitin for membranes” that labels and organizes autophagic membranes [41, 42]. LC3 is commonly detected in two forms: the cytosolic, non-lipidated LC3-I and the membrane-bound, phosphatidylethanolamine-conjugated LC3-II. Conversion of LC3-I to LC3-II is required for autophagosome membrane expansion and cargo recruitment, making this conjugation step a critical functional and biochemical step of autophagy. Through this membrane-embedded ubiquitin-like system, LC3 proteins govern cargo selection, phagophore elongation, and closure; mature autophagosomes ultimately fuse with lysosomes to form autolysosomes [42].

### Microautophagy

In microautophagy, cytosolic material is taken up directly through invaginations or protrusions of the vacuolar or lysosomal membrane. In mammalian cells, two mechanisms have been described: protrusions of the lysosomal limiting membrane and lysosomal invagination [42]. In hepatocytes, type 1 microautophagy has been visualized as arm- or flap-like protrusions of the lysosomal membrane that extend into the cytosol and close around bulk cytoplasm and small organelles. By contrast, lysosomal invagination underlies microlipophagy in mammalian hepatocytes, where inward budding of the lysosomal membrane engulfs entire lipid droplets into the lysosomal lumen for degradation. Because experimental monitoring remains technically challenging, this process is still less well characterized than macroautophagy [43].

### Chaperone-mediated autophagy (CMA)

**Fig. 1:**
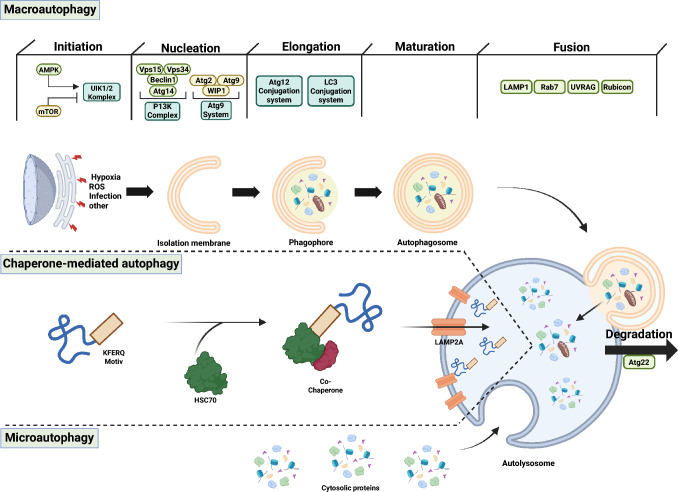
Core steps and molecular mediators of macroautophagy, chaperone-mediated autophagy and microautophagy. In macroautophagy (top), energy and nutrient status are sensed by the kinases AMPK (AMP-activated protein kinase) and mTORC1 (mechanistic target of rapamycin complex 1). AMPK is activated during energy stress and promotes autophagy, whereas mTORC1 is active in nutrient-rich conditions and suppresses it. Together, they control the ULK1/2-ATG13-FIP200/RB1CC1-ATG101 initiation complex, which acts as a central switch to start autophagosome formation. When AMPK is activated and mTORC1 is inhibited, ULK1 becomes active, recruiting membranes and downstream factors to the phagophore assembly site. During nucleation, the ULK1 complex activates the class III PI3K (PI3KC3) complex, consisting of VPS34, VPS15, Beclin 1 (BECN1) and ATG14L. This lipid kinase complex produces phosphatidylinositol-3-phosphate (PI3P) on selected membranes, which serves as a signal to recruit PI3P-binding proteins such as WIPI1 and ATG9-positive vesicles. Together, these factors shape the initial isolation membrane (phagophore). During elongation, two ubiquitin-like conjugation systems remodel the phagophore membrane. The ATG12-ATG5-ATG16L1 complex, assembled by the E1-like enzyme ATG7 and the E2-like enzyme ATG10, decorates the outer surface of the phagophore and acts as an E3-like factor. In parallel, LC3/ATG8 is first processed by the cysteine protease ATG4 and then conjugated to the membrane lipid phosphatidylethanolamine by ATG7 and the E2-like enzyme ATG3, generating LC3-II. Membrane-bound LC3-II both supports membrane expansion and provides a docking site for cargo receptors, thereby coupling membrane growth to cargo recruitment and closure of the autophagosome. Mature autophagosomes fuse with lysosomes. This maturation and fusion step is coordinated by small GTPases such as Rab7, which regulate vesicle transport and docking, by a Beclin 1-VPS34-VPS15-UVRAG complex that promotes fusion, and by lysosomal membrane proteins such as LAMP1. Fusion of the autophagosome with the lysosome produces an autolysosome, where cargo is degraded, and the resulting metabolites are exported back to the cytosol via lysosomal permeases (such as the yeast transporter Atg22 and related transporters in higher eukaryotes). In chaperone-mediated autophagy (middle), individual soluble cytosolic proteins carrying a KFERQ-like targeting motif are selectively recognized by the cytosolic chaperone HSC70 and co-chaperones. These complexes deliver the substrate proteins to the lysosomal receptor LAMP2A, where they are unfolded and translocated directly across the lysosomal membrane into the lumen for degradation. In microautophagy (bottom), cytosolic material is taken up directly by the lysosome through invagination, protrusion, and scission of the limiting membrane. This process allows bulk and, in some contexts, selective turnover of cytosolic proteins and organelle fragments independently of autophagosome formation. Created with BioRender.com

CMA is strictly selective for soluble cytosolic proteins bearing a KFERQ motif [44, 45]. HSC70/HSPA8 (heat shock protein family A [Hsp70] member 8) and co-chaperones recognize this motif, bind lysosomal-associated membrane protein 2A (LAMP2A) at the lysosomal membrane, unfold the substrate, and translocate it individually into the lumen for proteolytic degradation. LAMP2A transiently multimerizes to form a translocation complex and dissociates after transport [7]. The major autophagy pathways are visualized schematically in Fig. 1.

### Lipophagy: autophagic control of lipid droplets

Lipophagy represents a selective form of autophagy in which lipid droplets (LDs) play a central role. During this process, LDs are specifically degraded and function as supplementary lipid reservoirs, providing energy substrates for cellular metabolism and systemic energy balance [46, 47]. LDs are unique lipid-storing organelles composed primarily of triacylglycerols (TAGs) and sterol esters. These neutral lipids form a hydrophobic core that is enclosed by a monolayer of specific phospholipids and surface proteins, separating it from the cytoplasmic environment. The surface proteins are essential for maintaining LD functionality, stability, and intracellular mobility [48].

In mammalian cells, the lipid droplet monolayer is enriched in phosphatidylcholine, which plays an important role in LD stability and turnover [49]. The phospholipid composition of the LD membrane plays a crucial role in regulating LD synthesis, maturation, and degradation. Another critical determinant is droplet size, which defines the pathway of lipid catabolism [47, 50]. Smaller droplets are predominantly degraded by lipophagy, whereas larger droplets are mainly hydrolyzed by cytosolic lipases such as adipose triglyceride lipase (ATGL) [47, 51].

Beyond their function in lipid storage, LDs are also involved in the biosynthesis of membrane components, the generation of signaling ligands, and the storage of metabolic intermediates. This lipid buffering capacity offers a dual benefit: serving as an alternative energy source and protecting against excess free fatty acids (FFAs) and sterols, which could otherwise disrupt membrane integrity, signaling pathways, and overall metabolic balance [52]. For lipid homeostasis, the liver plays a crucial role in lipophagy. Impaired lipophagy results in defective LD degradation, leading to a significant buildup of triglycerides in hepatocytes, which ultimately promotes the development of fatty liver disease. Three main pathways of lipophagy reflect the broader autophagic system and are classified as macrolipophagy, microlipophagy, and chaperone-mediated lipophagy (CML). These pathways mainly differ in how the lipid droplet is delivered to and degraded by lysosomes.

In macrolipophagy, entire LDs are sequestered by autophagosomes and subsequently degraded following autophagosome-lysosome fusion, forming an autolysosome [53]. Microlipophagy is characterized by the direct engulfment of lipid droplets by lysosomes through membrane invagination, followed by lysosomal degradation. The size of the droplet is a decisive factor in determining the degradation route [47, 48]. Finally, chaperone-mediated lipophagy (CML) contributes to lipid-droplet turnover by selectively removing specific proteins from the LD surface. PLIN2 is a major LD coat protein that stabilizes lipid droplets and protects them from degradation. Its removal requires recognition by the chaperone HSC70/HSPA8 and delivery to the lysosomal receptor LAMP2A, thereby facilitating access of cytosolic lipases and autophagic machinery to the LD surface [54].

Over recent years, the concept of lipophagy has emerged as a central mechanism in lipid metabolism [55]. Although the molecular interfaces between individual lipoprotein fractions and the lipophagy machinery remain incompletely characterized, integrating these two systems provides a compelling mechanistic framework for understanding the interplay between extracellular lipid transport, intracellular lipid storage, and selective autophagy.

In particular, cholesterol-rich lipoproteins—including LDL, VLDL, and Apo(a)-containing particles—together with HDL as a cholesterol efflux partner, represent promising targets for elucidating how disturbances in lipid metabolism translate into cellular dysfunction. The concept of a direct interaction between lipophagy and lipoprotein pathways is highly attractive, as the classical lipoprotein fractions modulate intracellular lipid accumulation and thereby influence lipophagic activity. Triglyceride- and cholesterol-rich particles are internalized by macrophages and other cell types, via either scavenger receptors or ApoB/LDL receptor-dependent mechanisms, leading to the formation of cholesteryl ester (CE)-enriched lipid droplets [28]. Subsequently, lipophagy provides a pathway for breaking down these lipid stores, as LDs are moved to lysosomes via autophagosomes, where they are broken down by lysosomal acid lipase (LAL). The free cholesterol released then exits the cell through efflux transporters like ATP-binding cassette transporter (ABCA1), mainly to HDL [56]. HDL indirectly supports intracellular lipid clearance by accepting free cholesterol generated during LD mobilization and lysosomal lipid hydrolysis.

An imbalance in lipoprotein metabolism, such as elevated LDL/VLDL levels or reduced HDL-mediated efflux capacity, promotes excessive cellular lipid uptake and LD formation [57]. This overload can mechanically influence the lipophagic process. Studies show that prolonged exposure to oxidized LDL reduces lipophagic activity in endothelial cells [58]. When lipophagy is impaired, lipid droplets persist, cholesterol efflux is disrupted, and cellular stress or apoptosis increases, which promotes lipid-related diseases like atherosclerosis [29]. Overall, this creates a cycle: high lipoprotein uptake leads to LD formation, which triggers lipophagy and HDL-mediated cholesterol removal. When this cycle becomes dysfunctional, harmful lipid accumulation occurs, damaging cellular and organ functions.

Together, these mechanisms illustrate that lipophagy forms a central interface between extracellular lipoprotein metabolism and intracellular lipid clearance. However, when lipid influx chronically exceeds lysosomal and autophagic capacity, this adaptive system can shift into a maladaptive state characterized by impaired autophagic flux, lysosomal stress, mitochondrial dysfunction, and inflammatory activation.

## How lipid overload disrupts autophagic flux

Lipophagy initially acts as an adaptive mechanism that protects cells from lipotoxicity by mobilizing lipid droplets and supporting lysosomal lipid degradation [53, 55]. However, when lipid influx chronically exceeds the capacity of lipid storage, lipolysis, and lysosomal clearance, this compensatory system can become maladaptive [59]. Under these conditions, autophagosome formation may still be induced, but efficient cargo degradation becomes limited, resulting in impaired autophagic flux rather than productive autophagy [59]. Therefore, accumulation of autophagy markers such as LC3-II or SQSTM1/p62 should be interpreted cautiously, as it may reflect either increased autophagosome formation or defective lysosomal degradation [60, 61].

A central mechanism linking lipid overload to autophagic flux impairment is lysosomal stress [59]. Lysosomes are responsible not only the terminal degradative compartment of autophagy, but also for cholesterol trafficking, lipid hydrolysis, nutrient sensing, and metabolic signaling [59]. Excess cholesterol, oxidized lipids, and lipid-derived damage products can disturb lysosomal membrane stability, acidification, and hydrolase activity [59, 62]. This compromises autophagosome-lysosome fusion and autolysosomal degradation, thereby promoting the accumulation of lipid droplets, damaged organelles, and undegraded cargo [59, 62]. In macrophages and vascular cells, such lysosomal dysfunction may be particularly relevant because uptake of modified lipoproteins imposes a sustained degradative load and contributes to foam-cell formation, oxidative stress, and inflammatory activation [56, 62].

Lipid overload also intersects with endoplasmic reticulum stress and the unfolded protein response [59]. Excess saturated fatty acids, cholesterol accumulation, and altered membrane composition disturb ER homeostasis, protein folding, and calcium handling. While acute ER stress can activate autophagy as a compensatory response, chronic ER stress may impair autophagic flux by disturbing autophagosome maturation, lysosomal function, and nutrient-sensing pathways such as AMPK and mTORC1. In this setting, autophagy shifts from a protective recycling program to an insufficient stress response, thereby allowing lipid droplets, damaged membranes, and dysfunctional organelles to accumulate [59].

Mitochondrial dysfunction represents another key consequence of lipid-induced autophagy impairment. Excess lipid species and defective lysosomal clearance promote mitochondrial damage, increased mitochondrial ROS production, and impaired mitophagy [59, 63]. Damaged mitochondria further amplify oxidative stress and release mitochondrial danger signals that activate inflammatory pathways. Thus, lipid overload, lysosomal dysfunction, and defective mitophagy form a self-reinforcing loop in which impaired organelle quality control drives persistent cellular stress. This loop is particularly important because mitochondrial ROS and lysosomal damage are both recognized triggers of NLRP3 inflammasome activation, linking autophagic flux failure to chronic inflammatory signaling [63].

In addition to lipid overload, other metabolic stressors may amplify autophagic failure through convergent stress pathways. Hyperhomocysteinemia, for example, has been linked to ER stress, oxidative stress, mitochondrial dysfunction, lysosomal impairment, and reduced autophagic flux [64, 65]. Elevated homocysteine can promote protein misfolding and unfolded protein response activation, while increased ROS production and mitochondrial injury further burden the autophagy-lysosome system [64, 65]. Hyperhomocysteinemia should therefore be understood as a metabolic modifier that reinforces lipid-induced lysosomal and mitochondrial stress rather than as an independent thematic axis.

Together, these mechanisms explain how chronic lipid overload can convert autophagy from an adaptive quality-control pathway into a dysfunctional and incomplete stress response. Once lysosomal degradation, mitochondrial quality control, and ER homeostasis are simultaneously compromised, cells accumulate damaged organelles, lipid droplets, oxidized lipids, and inflammatory signals [59, 63].

## Autophagy in inflammatory resolution and inflammasome restraint

Beyond its role in intracellular housekeeping, autophagy is an important determinant of inflammatory resolution. A central function in this context is the support of phagocytic cargo processing after uptake of dying cells, pathogens, or cellular debris. Autophagy-related proteins contribute to phagosome maturation and to non-canonical LC3-associated phagocytosis (LAP), a pathway in which elements of the autophagy machinery are recruited to single-membrane phagosomes to facilitate lysosomal degradation of engulfed material. This is particularly relevant during efferocytosis, the clearance of apoptotic cells, because efficient degradation of apoptotic cell remnants is essential for maintaining tissue homeostasis and for preventing secondary necrosis and release of proinflammatory contents [66–68].

The contribution of autophagy to phagosome maturation is not merely degradative, but also immunoregulatory. By promoting efficient lysosomal processing of engulfed cargo, LAP and related autophagy-dependent pathways favor an anti-inflammatory clearance program and limit the persistence of damage-associated molecular patterns (DAMPs). When these pathways are impaired, engulfed apoptotic material is processed less efficiently, macrophage efferocytosis and post-engulfment cargo degradation become less efficient, and inflammatory lesions are more likely to progress toward unresolved necrosis. In advanced atherosclerosis, for example, macrophage autophagy has been shown to play a protective role by limiting oxidative stress, apoptosis, and plaque necrosis in part through preserving effective clearance mechanisms [67, 68].

Autophagy also restrains inflammatory signaling more directly. One mechanism is the autophagic degradation of pro-IL-1β or inflammasome-related components, which reduces the pool of substrate available for maturation and secretion [69]. In addition, autophagy limits the generation of cleaved IL-1β by suppressing inflammasome assembly and caspase-1-dependent processing upstream [69–71]. In parallel, autophagy preserves mitochondrial integrity and promotes removal of damaged mitochondria, thereby limiting mitochondrial ROS generation and mitochondrial DNA release, two major triggers of NLRP3 inflammasome activation [70]. Consistent with this, loss of key autophagy proteins such as ATG16L1 enhances endotoxin-induced IL-1β production, whereas intact autophagic function suppresses excessive inflammasome activity [71]. Emerging evidence further suggests that selective autophagic pathways may also reduce pyroptotic execution by promoting degradation of gasdermin D (GSDMD), including the pore-forming cleaved form [72]. By limiting both cleaved IL-1β generation and cleaved GSDMD-dependent membrane pore formation, autophagy helps restrain pyroptosis and thereby dampens inflammatory tissue injury [69–72].Together, these observations position autophagy as an active regulator of inflammatory resolution rather than a passive quality-control pathway. When autophagic clearance fails, damaged organelles, apoptotic material, and proinflammatory intermediates accumulate simultaneously. The result is a feed-forward state characterized by defective efferocytosis, delayed phagolysosomal processing, persistent inflammasome competence, and sustained cytokine production. This framework is highly relevant to chronic inflammatory disorders driven by metabolic stress, including atherosclerosis and MASLD/MASH, in which impaired clearance and unresolved inflammation reinforce tissue injury and disease progression [67–71].

## Disease-specific consequences of autophagy failure

### Cardiovascular disease

Lipids regulate autophagy at multiple stages—from membrane nucleation and autophagosome growth to cargo selectivity and lysosomal turnover, while autophagy safeguards lipid quality control, clearing damaged membranes and lipid droplets [73]. Atherosclerosis, a major contributor to the high mortality associated with cardiovascular disease, arises in part from impaired autophagy. Traditionally viewed primarily as a disorder of lipid accumulation, atherosclerosis is now understood as a chronic inflammatory disease in which lipid metabolism impacts the buildup of plaques, causing a higher risk of myocardial infarction, and strokes [74]. Atherosclerosis is multifactorially driven by the immune system, inflammation, lipid metabolism and the progression of the disease is strongly influenced by the underlying autophagic mechanisms and pathways governing these interactions [74]. In the early stages of atherosclerosis, autophagy inhibits the development of plaques, removing damaged organelles and reducing foam cell formation, but as the disease progresses, autophagy gets progressively impaired [75–77]. Under sustained lipid stress, impaired autophagy leads to the accumulation of damaged organelles, oxidized lipids, and inflammatory cargo, thereby promoting monocyte recruitment to the vascular wall. [78, 79]. The normally circulating monocytes are recruited to the arterial wall, where they differentiate into macrophages, which in turn clear and internalize modified lipoproteins. When cholesterol efflux to HDL is insufficient, these macrophages accumulate lipid droplets and transform into foam cells, promoting the growth of atherosclerotic plaques [56, 80]. Foam-cell apoptosis and defective efferocytosis promote accumulation of apoptotic and necrotic material, leading to necrotic core formation and plaque instability [81, 82]. Furthermore, inflammatory signaling amplifies arterial wall injury and contributes to plaque progression and instability [79, 83].

Increased and sustained expression of autophagy marker proteins Beclin1 and LC3-II induced by ox-LDL leads to elevated calcium ion concentration in the plasma membrane, induces endoplasmic reticulum stress, and activates pro-apoptotic factors, resulting in endothelial cell apoptosis. These apoptotic cells enhance platelet adhesion and promote thrombus formation after plaque rupture, linking impaired autophagy to myocardial infarction or strokes [13]. Severe oxidative stress promotes lipofuscin/ceroid-like material accumulation, which can further impair lysosomal hydrolase activity and autophagic degradation, disrupts lysosomal hydrolase function, and, in turn, leads to apoptosis and mitochondrial accumulation. This promotes increased oxidative stress and waxy pigmentation production, leading to a vicious circle that exacerbates cell death [84, 85]. Oxidative stress additionally induces mitochondrial damage, which can be cleared through mitophagy activation via the PINK/Parkin pathway. However, in advanced atherosclerosis, the mechanism becomes inefficient, further accumulating damaged mitochondria and elevating the oxidative stress levels [86]. These findings are consistent with the broader interpretation that increased autophagy marker levels do not necessarily indicate effective autophagic degradation; rather, in endothelial pathology, impaired or blocked autophagic flux may contribute to cellular dysfunction [61, 87].

### Metabolic disease

Autophagy in obesity is highly context and cell-type-dependent. White adipose tissue (WAT) explants, containing adipocytes and fibroblasts, show elevated levels of autophagic markers in total, but the cell types differ remarkably. The mature adipocytes themselves show lower autophagic flux, reflected by reduced LC3-II accumulation [88–90]. Autophagic markers must be interpreted with caution, because their accumulation may reflect either increased autophagosome formation or impaired lysosomal degradation. Therefore, assessment of autophagic flux is essential for correct mechanistic interpretation [61]. Macrophages display decreased autophagic flux, infiltrating WAT during obesity, impacting insulin sensitivity, enhanced activation of inflammatory signaling pathways and defective clearance of damaged organelles [91, 92]. Additionally, other modifications occur, such as reduced expression of insulin receptor substrate (IRS-1) expression, impacting the PI 3-kinase, one of the key signal transducers in insulin-stimulated glucose uptake [93, 94]. Through insulin resistance, hyperglycemia, hypertension, dyslipidemia, hyperuricemia and endothelial dysfunction are caused. Continuously increasing insulin resistance will lead to Type 2 Diabetes Mellitus, metabolic syndrome, or metabolic dysfunction-associated steatotic liver disease, MASLD [95]. MASLD and its progressive form, metabolic dysfunction-associated steatohepatitis (MASH), represent clinical phenotypes of hepatic autophagy dysfunction, initiated and perpetuated by chronic systemic inflammation and metabolic stress [7, 8]. Lipophagy is critical in maintaining hepatic lipid homeostasis in MASLD/MASH patients by degrading lipid droplets and shuttling free fatty acids for mitochondrial oxidation [29, 96]. While autophagy is initially upregulated in response to early lipid overload, chronic nutrient excess (by Western-style diet) and lipotoxicity impair autophagic flux through the AMPK-mTOR axis, leading to the accumulation of dysfunctional organelles and lipid droplets, weaken lipophagic capacity and accelerate hepatic steatosis [96]. Cholesterol overload has been linked to lysosomal dysfunction and NLRP3 inflammasome activation, mechanisms that may also be relevant to MASLD/MASH progression [97]. Impaired lipophagy also drives overproduction of VLDL, promoting atherogenic dyslipidemia and increasing cardiovascular risk [98]. Altered VLDL metabolism may further intensify intracellular lipid loading ans systemic dyslipidemia, thereby reinforcing lipotoxic stress [98, 99]. Epidemiological data confirm that both MASLD and MASH independently elevate the risk for cardiovascular disease (CVD), underscoring hepatic autophagy dysfunction as a mechanistic link between metabolic liver disease and systemic inflammation [96]. Furthermore, lifestyle factors such as alcohol further disturb lipid metabolism through comparable pathogenic mechanisms [47]. Disease trajectory is further modified by sex, age, and hormonal status, but these factors are beyond the main mechanistic focus of this review [100–102].

The impairment of hepatic autophagy extends beyond the liver, as dysfunctional hepatocytes release damage-associated molecular patterns (DAMPs) and additional proinflammatory cytokines that amplify hepatic and systemic inflammation [103]. In metabolic liver disease, chronic lipid overload in the setting of impaired autophagy and lysosomal dysfunction may also increase susceptibility to ferroptotic membrane damage. Under physiological conditions, lipophagy, mitophagy, and ferritinophagy buffer metabolic stress by removing ROS-producing mitochondria, controlling the cellular iron pool, and limiting the accumulation of peroxidation-prone membranes; when autophagy is impaired, this protective capacity is lost, resulting in increased ferroptotic vulnerability [104–106]. Of particular importance is NCOA4-mediated ferritinophagy, as its dysregulation can expand the redox-active iron pool and thereby enhance lipid peroxidation [105, 107]. In addition, lysosomal dysfunction under conditions of oxidative stress and lipid overload further aggravates mitochondrial injury and oxidative damage. In the context of MASLD/MASH, iron overload, lipid peroxidation, inflammatory cell death, and defective autophagy therefore converge into a self-reinforcing pathogenic mechanism [106, 108].

Beyond metabolic and inflammatory toxicity, defective autophagy and lipid overload increase ROS and lipid peroxidation, leading to intracellular accumulation of mutagens. Reactive aldehydes form highly mutagenic DNA adducts that are detected in MASLD and MASH patients, linking impaired autophagy to genomic instability and tumorigenesis [109, 110].

These mechanisms together enhance tissue oxidative stress, promote vascular foam cell formation, and compromise cellular clearance systems, ultimately contributing to disease progression rather than conferring metabolic benefits. Therefore, therapeutic strategies aimed at restoring autophagic competence not only improve hepatic function but also provide significant cardiovascular protection by interrupting these maladaptive metabolic-inflammatory cycles.

### Neurocognitive disease

Defective autophagy and lipid overload lead to a reinforcing loop that can accelerate multiple neurodegenerative diseases [73, 111, 112]. When lipid regulation and autophagic lipid clearance are uncoupled, lipid droplets accumulate in neurons and glial cells, leading to impaired lysosomal flux and amplified neuroinflammatory signaling [73, 111, 112]. In Alzheimer disease (AD), this is driven by ceramide dysregulation, lipid-droplet-accumulating microglia, and APP-βCTF-mediated lysosomal dysfunction, all of which promote autophagic vacuole buildup, amyloid pathology, synaptic injury, and cognitive decline [111, 113, 114]. Genetic factors like APOE4 amplify this state by promoting maladaptive lipid handling even further and increasing lipotoxic stress [115, 116]. In Parkinson disease (PD), lipid droplets can act as nucleation sites for α-synuclein aggregation, while modified α-synuclein inhibits chaperone-mediated autophagy and disrupts macroautophagy through ATG9 and RAB1A, thereby impairing lysosomal clearance and lipophagy [117–120]. GBA1 loss-of-function further alters lysosomal lipid composition and increases α-synuclein accumulation [121]. In vascular dementia (VaD), autophagy defects in the neurovascular unit contribute to endothelial dysfunction, while impaired astrocytic and microglial lipophagy under ischemic stress promotes lipid droplet accumulation, oxidized lipid stress, defective debris clearance, and white matter injury [122–125]. Dysregulated LD dynamics can transform astrocytes from neuroprotective to pro-inflammatory states that exacerbate white matter injury typical of VaD [124].

Across Alzheimer disease, Parkinson disease, and vascular dementia, impaired autophagy—especially defective lipophagy—disrupts neuronal and glial lipid homeostasis, fosters lipid-droplet accumulation, and compromises lysosomal flux. These failures in lipid and proteostasis amplify neuroinflammation, synaptic dysfunction, and cognitive decline, with disease-specific factors such as ceramide dysregulation and APP-βCTF in AD, α-synuclein pathology and GBA1 loss-of-function in PD, and endothelial or astroglial autophagy defects at the neurovascular unit in VaD.

### Emerging disease contexts

Beyond classical cardiometabolic disease, emerging evidence suggests that defective autophagic clearance may also contribute to disease states characterized by disrupted temporal regulation. Autophagic flux is closely coupled to circadian timing cues such as feeding-fasting cycles, sleep, and light–dark exposure, and this coordination helps align nutrient sensing, lysosomal competence, and mitochondrial quality control with predictable metabolic demand [126–130]. When circadian organization is lost, mitophagic efficiency declines, inflammasome activity may become prolonged, and immunometabolic flexibility is reduced [127–130].

Another related concept has emerged in post-viral syndromes such as PASC, in which acute viral injury appears to disrupt autophagic and mitochondrial homeostasis beyond the period of active infection. SARS-CoV-2 interferes with autophagosome maturation and is associated with mitochondrial dysfunction and impaired mitophagy, thereby favoring persistent inflammatory signaling [35, 131–134]. EBV reactivation may represent an additional post-infectious modifier, as EBV also interacts with autophagic machinery and has been reported more frequently in COVID-19 and post-COVID cohorts [135–137].

Although the direct contribution of lipid overload is less well established than in atherosclerosis or MASLD/MASH, both circadian disruption and post-viral syndromes support the broader concept that failure to restore autophagic homeostasis after metabolic or infectious stress can promote chronic inflammation, fatigue, and multisystem dysfunction [127, 138, 139].

## Therapeutic and translational perspectives

As previously described, circulating cholesterol fractions (VLDL, LDL, oxLDL, Lp(a)) impact the autophagic flux and promote chronic inflammatory diseases through metabolic overload. Although HDL promotes cholesterol efflux, the remaining fractions contribute directly to autophagy dysfunction by impairing autophagosome maturation, promoting oxidative stress and disrupting lysosomal pathways. Through associated processes like insulin resistance, atherosclerosis and MASLD/MASH, a self-reinforcing loop of lipid overload, impaired autophagy and chronic inflammation is established [73, 140, 141]. Autophagy can be conceptualized as a modular network: initiation, autophagosome maturation, lysosomal capacity, organellophagy (mitophagy, lipophagy, iron handling). This implies that lipid driven diseases may arise from distinct failures in specific nodes, requiring context and pathway specific intervention [141]. Therapeutic strategies to reduce lipid load and improve autophagy therefore represent approaches to break the inflammatory loop and improve quality of life [140].

### Lifestyle and metabolic unloading

The primary therapeutic strategy for improving patient health is exercise and behavioral intervention, which also act as modulators of autophagy. In preclinical models, exercise improved lipid handling and lipid clearance [142]. Moderate exercise training has also been identified as an effective strategy to ameliorate MASLD progression, leading to increased mitochondrial function and lipid oxidation [143]. In addition, dietary modulation through intermittent fasting may represent another clinically relevant autophagy-inducing strategy, reducing lipotoxicity and supporting periodic activation of autophagic flux [144]. Chronobiologic alignment may provide an additional approach, as time-restricted eating can leverage endogenous circadian oscillations in hepatic and immune autophagy pathways [145].

### Pharmacologic therapies

Effective pharmacological intervention for autophagy collapse requires a “twin approach”; reducing lipotoxicity and restoring autophagic flux on one hand, and resolving inflammatory load on the other, because targeting only one arm of this pathogenic loop may not resolve the underlying cause of dysregulation [141].

For inducing autophagic functions, the AMPK-mTOR pathway represents a central therapeutic target.

Pharmacological inhibition of mTORC1 by rapamycin can activate autophagy, although precise tuning is required since excessive activation may lead to unwanted catabolic effects [146, 147]. Metformin activates autophagy as well through AMPK, but off-target and context dependent effects must be considered [32, 33]. As part of the AMPK-mTOR pathway, Beclin-1 and class III PI3K is another direct target for improving autophagosome biogenesis. Tat-Beclin-1, a Beclin-1 activating peptide, enhances hepatic autophagy and ameliorates MASLD-like phenotypes [148]. Another transcriptional node is TFEB, transcription factor EB. Recognized as a major transcriptional regulator of autophagy, targeted regulation may improve metabolic disorders, neurodegenerative diseases and inflammatory disease. Beyond AMPK-mTOR, SIRT1-dependent interventions may provide an additional regulatory layer by coupling nutrient sensing to deacetylation-driven autophagy and mitophagy, thereby supporting metabolic resilience [149].

Furthermore, selective pathways of autophagy are traceable in lipid-driven disease. Lipophagy therapies improve hepatic steatosis and systemic insulin sensitivity, thereby reducing the substrate burden that precipitates lipotoxic autophagy collapse. In this context, semaglutide showed beneficial changes in metabolic dysfunction-associated steatohepatitis, while tirzepatide improved MASH resolution without worsening fibrosis [150, 151]. Likewise, Mitophagy enhancement can reduce ROS-generating mitochondria and dampen inflammatory signaling. In the context of combined lipid overload and mitochondrial dysfunction, urolithin A has shown promising results after a randomized clinical study [152].

Lowering atherogenic lipoproteins, primarily LDL cholesterol and Lp(a), is an essential strategy of reducing cardiovascular risk and, within the framework of this review, for reducing the upstream lipid burden that drives autophagic dysfunction. Current treatment typically begin with tolerated statin therapy, with add-on non-statin agents when LDL cholesterol remains above target [153]. Beyond lipid lowering, experimental studies suggest that statins may modulate autophagy-related pathways, including AMPK/mTORC1 signaling [154]. In macrophages, atorvastatin has shown to increase autophagic flux, while reducing inflammatory signaling and foam-cell formation [155]. If cholesterol remains above target despite statin therapy, PCSK9 (proprotein convertase subtilisin-kexin type 9) inhibitors such as evolocumab or alirocumab can achieve a significant reduction in major adverse cardiovascular events [156, 157]. In hepatocytes, PCSK9 interacts with apoB and prevents its degradation, thereby contributing to increased apo-B-lipoprotein burden [158]. Conversely, inhibition of PCSK9 increases autophagic flux, reduces oxidative stress and inflammatory signals, and combines the previously mentioned “twin approach” of reducing inflammation while increasing autophagy and reducing lipotoxicity [159]. Beyond established approaches, newer lipid-centered therapies may indirectly improve autophagy and lysosomal function by reducing lipid burden. Olezarsen inhibits ApoC-III, reducing triglycerides in hypertriglyceridemia, and solbinsiran reduces atherogenic lipoproteins by silencing ANGPTL3 (Angiopoietin Like 3) in mixed dyslipidemia [160, 161]. For long-term LDL-lowering, inclisiran represents an additional siRNA-based strategy targeting PCSK9 [162].

### Biomarker

To address these specific aspects, biomarkers must be identified for each target. While cholesterol and inflammatory markers are well-established diagnostic standards, biomarkers for autophagy are less thoroughly studied. Currently, there is no single universal biomarker for autophagy because the dynamic nature of "flux” means that static measurements can be misleading. Instead, it is better to use paired readouts that reflect cargo processing, such as LC3 turnover and SQSTM1/p62 degradation [61]. Additionally, at the pathway level, TFEB-driven CLEAR-network transcripts serve as a transcriptional indicator of autophagy-lysosome capacity [163]. In a lipid-focused context, combined readouts that integrate autophagy markers with lipid-droplet or lysosomal markers may help identify interventions that restore hepatic lipid turnover [7]. In addition, markers like ceramides and phospholipid ratios highlight downstream metabolic effects of impaired autophagy [8]. Exploratory studies suggest that Beclin-1 and selected autophagy-related non-coding RNAs may have future biomarker potential, although these approaches still require substantial validation [164, 165].

### Emerging therapeutic contexts

Emerging evidence suggests that autophagy dysregulation may also contribute to clinical conditions characterized by incomplete recovery after acute stress and by disturbed temporal organization of metabolism. In this context, post-viral syndromes such as Long COVID and related fatigue syndromes have gained increasing attention, as they appear to involve persistent alterations in mitochondrial homeostasis, inflammatory signaling, and autophagy-related pathways. Supporting the therapeutic relevance of this concept, a recent study in patients with ME/CFS reported that once-weekly low-dose rapamycin led to clinically meaningful improvements in fatigue, post-exertional malaise, and orthostatic intolerance, accompanied by increased plasma BECLIN-1 levels, consistent with restoration of autophagy signaling [147]. Because post-exertional symptom exacerbation and other clinical features overlap substantially between ME/CFS and Long COVID, these findings suggest that mTOR-dependent autophagy impairment may represent a shared mechanism and support autophagy-modulating strategies as a plausible therapeutic avenue in post-viral syndromes [147].

In parallel, nutritional and chronobiologic interventions may provide additional therapeutic entry points in these emerging contexts. A trial in individuals with Long COVID reported that intensified intermittent fasting produced greater improvements in global symptom burden and symptom count than milder time-restricted eating alone [166]. Although autophagy and lipophagy markers were not directly assessed, these findings are consistent with the hypothesis that periodic, prolonged fasting may help restore metabolic and organellar homeostasis in post-viral syndromes [166]. Chronobiologic alignment may further complement this approach, as time-restricted eating can reinforce endogenous circadian oscillations in hepatic and immune autophagy pathways [145]. Melatonin has likewise been discussed as a chronobiotic and cytoprotective signal with anti-inflammatory and redox-modulatory properties that could synergize with autophagy-restorative strategies in chronic inflammatory disease [167]. Together, these observations suggest that post-viral persistence and circadian disruption may represent emerging contexts in which defective autophagic recovery contributes to ongoing symptoms and may offer new therapeutic entry points.

As immunometabolism progresses, it becomes clearer that immune function is closely linked to the cell’s metabolic state and depends on effective autophagy. Understanding how specific types of lipids influence autophagy could lead to targeted therapies to address chronic inflammation and improve cell resilience.

## Conclusion

Lipid dysregulation and impaired autophagy are tightly interconnected processes that together promote chronic inflammatory disease. Under physiological conditions, lipophagy, mitophagy, and lysosomal degradation maintain lipid and organelle homeostasis. However, when chronic lipid overload exceeds autophagic and lysosomal capacity, this adaptive system shifts toward impaired autophagic flux, lipid-droplet accumulation, mitochondrial dysfunction, oxidative stress, and persistent inflammatory signaling (Table 2). In this setting, autophagy failure not only compromises intracellular lipid handling, but also weakens inflammatory resolution by impairing efferocytosis, phagolysosomal processing, and inflammasome restraint. This transition provides a mechanistic link between disturbed lipoprotein metabolism and unresolved inflammation.

**Table 2:** Systemic modifiers of autophagy and their consequences

Type	Factor/context	Effect on autophagy	Consequence	Reference
Inflammation and immune regulation	Chronic inflammation	Pro-inflammatory cytokine signaling and NLRP3 activation impair autophagic flux and clearance of damaged organelles	Sustained metabolic dysfunction, plaque progression, organ damage	[18, 35, 74, 141]
Inflammation and immune regulation	Defective immunoregulation	Reduced autophagy in immune cells alters cytokine control and macrophage inflammatory polarization and weakens inflammatory resolution	Chronic low-grade inflammation, impaired clearance responses, plaque vulnerability	[35, 68, 75, 92, 141]
Inflammation and immune regulation	Mitochondrial dysfunction & oxidative stress	Impaired mitophagy leads to accumulation of damaged mitochondria and excess ROS, promoting NF-κB- and NLRP3-linked inflammatory signaling	Chronic inflammation, tissue injury, increased susceptibility to cell death	[63, 70, 84, 86, 103]
Inflammation and immune regulation	Persistent viral infections (EBV, SARS-CoV-2/PASC)	Viral proteins exploit or block autophagic machinery and impair autophagosome maturation and mitophagy	Chronic inflammatory state, post-acute sequelae, fatigue and neurocognitive symptoms	[131, 134–139]
Metabolic and nutrient-sensing pathways	mTORC1 hyperactivation	Chronic nutrient excess activates mTORC1 and suppresses ULK1-dependent autophagy initiation	Metabolic overload, impaired clearance of lipids and damaged organelles	[32, 33, 38]
Metabolic and nutrient-sensing pathways	AMPK inhibition	Reduced AMPK activity limits autophagy induction and metabolic adaptation	Lipotoxicity, reduced energy adaptation, accumulation of metabolic waste	[32, 33]
Metabolic and nutrient-sensing pathways	ATG3/ATG5 deficiency	Loss of essential autophagy machinery blocks autophagosome formation	Collapse of autophagic clearance, impaired removal of protein aggregates and organelles	[37, 41]
Metabolic and nutrient-sensing pathways	ER stress	Chronic unfolded protein response and disturbed calcium handling impair autophagic flux and lysosomal function	Accumulation of misfolded proteins and dysfunctional organelles, promotion of cell-death signaling	[36, 59, 64]
Metabolic and nutrient-sensing pathways	Lysosomal dysfunction (acidification, fusion)	Impaired lysosomal acidification and autophagosome-lysosome fusion reduce degradative capacity	Waste accumulation, persistent inflammation, impaired host defense	[59, 62, 141]
Lipid metabolism and lipoproteins	VLDL ↑	Triglyceride-rich lipoprotein overload increases intracellular lipid burden and promotes lipotoxic and ER/lysosomal stress	Lipotoxicity, atherogenic dyslipidemia, contribution to MASLD progression	[15, 25, 98, 99]
Lipid metabolism and lipoproteins	LDL/oxLDL ↑	Chronic oxLDL exposure promotes lysosomal lipid accumulation, ER stress, and impaired autophagic flux in vascular cells and macrophages	Foam-cell formation, necrotic core expansion, atherosclerosis progression	[13, 24, 56, 58, 78, 79]
Lipid metabolism and lipoproteins	HDL ↑	Promotes cholesterol efflux and supports lipid clearance; may preserve autophagic/lipophagic competence in lipid-stressed cells	Reduced inflammation, improved reverse cholesterol transport, plaque stabilization	[13–17, 28, 56]
Lipid metabolism and lipoproteins	Lp(a) ↑	Pro-inflammatory Apo(a)-containing lipoprotein associated with oxidized phospholipids and vascular inflammation; the direct autophagy link remains incompletely defined	Endothelial dysfunction, plaque vulnerability, thrombotic risk	[26, 27, 74]
Metabolic stressors and comorbidities	Homocysteine ↑ (HHcy)	Promotes ER stress, oxidative stress, inflammasome activation, and reduced autophagic flux	Endothelial injury, pyroptotic signaling, elevated cardio- and cerebrovascular risk	[64, 65]
Metabolic stressors and comorbidities	MASLD/MASH	Early adaptive lipophagy is followed by impaired autophagic flux, lysosomal lipid stress, and inflammatory activation	Hepatic triglyceride accumulation, VLDL overproduction, systemic inflammation, increased CVD risk	[7, 8, 15, 29, 59, 96, 98]
Metabolic stressors and comorbidities	Obesity and WAT inflammation	Cell-type-specific impairment of autophagic flux in adipocytes and adipose macrophages with enhanced inflammatory signaling	Insulin resistance, systemic low-grade inflammation, progression to T2DM and MASLD/MASH	[88–92, 95]
Ferroptosis and organelle stress	Iron overload, ferritinophagy and lysosomal defects	Disturbed ferritinophagy and lysosomal damage increase redox-active iron and lipid peroxidation under impaired autophagic control	Ferroptotic vulnerability in macrophages, endothelial cells, and hepatocytes; plaque and MASH progression	[84, 105–109]
Neurodegeneration and neurovascular injury	Alzheimer disease, Parkinson disease, vascular dementia	Lipid-autophagy defects, CMA impairment, lysosomal dysfunction, and glial lipid-droplet accumulation	Neuroinflammation, synaptic failure, white-matter injury, cognitive decline	[73, 111–125]
Circadian and chronobiologic control	Circadian disruption (shift work, irregular feeding, post-infectious rhythm disturbance)	Desynchronizes clock-controlled autophagy/mitophagy and alters time-of-day gating of inflammasome activity	Persistent metabolic and immune activation, increased cardiometabolic and post-infectious disease risk	[126–130]
Lifestyle and environmental factors	Intermittent fasting / time-restricted eating	Cyclic AMPK activation, relative mTORC1 inhibition, and reinforcement of circadian metabolic-autophagy coupling	Improved metabolic flexibility, reduced hepatic steatosis and inflammation; symptom improvement in selected post-viral cohorts	[33, 144, 145, 166]
Lifestyle and environmental factors	Exercise (moderate, regular)	Promotes mitochondrial function, lipid handling, and autophagy/lipophagy-related adaptation in metabolic tissues	Improved insulin sensitivity, hepatic lipid handling, vascular function, and physical resilience	[142, 143]
Lifestyle and environmental factors	Alcohol consumption	Alcohol-related liver injury is associated with hepatic steatosis and oxidative stress and may secondarily impair lipophagy-related homeostasis	Worsening steatotic liver disease and fibrogenic progression	[47, 49]
Lifestyle and environmental factors	Synthetic hormones (estrogens, progestogens; contraception, HRT)	Indirect modulation of hepatic lipid metabolism and disease trajectory rather than direct autophagy-specific effects	Hormone-regimen-dependent differences in MASLD prevalence and fibrosis risk	[100–102]
Therapeutic modulators of autophagy	mTOR/AMPK-related interventions	Modulate nutrient-sensing pathways and can enhance autophagic flux in metabolically stressed cells	Improved lipid handling and organelle quality control; potential symptom improvement in selected fatigue syndromes	[32, 33, 146, 147]
Therapeutic modulators of autophagy	TFEB-directed strategies	Enhance autophagy-lysosome biogenesis and lysosomal transcriptional programs	Restoration of autophagic reserve and improvement of steatotic phenotypes in preclinical settings	[148, 163]
Therapeutic modulators of autophagy	SIRT1 / mitophagy modulators (including urolithin A)	Support SIRT1-dependent autophagy/mitophagy and mitochondrial quality control	Reduced ROS burden and improved cellular resilience	[149, 152]
Therapeutic modulators of autophagy	Lipid-lowering drugs with autophagy-related effects (statins, PCSK9 inhibitors, ApoC-III/ANGPTL3-targeting agents)	Reduce atherogenic lipid burden; selected agents may additionally improve autophagy-related signaling in macrophages or hepatocytes	Reduced cardiovascular events, improved plaque stability, indirect relief of lipotoxic autophagy stress	[153–162]

This framework is particularly relevant to atherosclerosis and MASLD/MASH, where defective autophagy links metabolic overload to unresolved inflammation, organ dysfunction, and disease progression. Related mechanisms also extend to neurocognitive disorders and to emerging contexts such as post-viral syndromes and circadian disruption, although in these settings the direct role of lipid overload is less well defined.

Therapeutically, the lipid-autophagy-inflammation axis supports a multi-level intervention strategy. Reducing lipid burden, improving metabolic flexibility, restoring lysosomal-autophagic flux, and limiting inflammatory signaling may together interrupt self-reinforcing disease loops. Future studies should move beyond static autophagy markers and incorporate flux-based readouts, lipidomic profiling, lysosomal competence measures, and disease-specific patient stratification. Such approaches may enable more precise targeting of autophagy failure and help translate mechanistic insights into therapies for chronic metabolic and inflammatory disease.

## Data Availability

No datasets were generated or analysed during the current study.

## Abbreviations

ABCA1: ATP-binding cassette transporter A1
AD: Alzheimer disease
AMPK: AMP-activated protein kinase
ANGPTL3: Angiopoietin-like 3
Apo(a): Apolipoprotein(a)
ApoB-100: Apolipoprotein B-100
ApoC-III: Apolipoprotein C-III
APOE4: Apolipoprotein E 4
APP: Amyloid precursor protein
APP-βCTF: APP β-carboxy-terminal fragment
ATG: Autophagy-related gene
CLEAR: Coordinated lysosomal expression and regulation network
CMA: Chaperone-mediated autophagy
EBV: Epstein–Barr virus
ER: Endoplasmic reticulum
GBA1: Glucocerebrosidase 1
HDL: High-density lipoprotein
IDL: Intermediate-density lipoprotein
LAMP1: Lysosome-associated membrane protein 1
LAMP2A: Lysosome-associated membrane protein 2A
LC3: Microtubule-associated protein 1A/1B-light chain 3
LC3-II: Lipidated LC3
LD: Lipid droplet
LDL: Low-density lipoprotein
Lp(a): Lipoprotein(a)
MASLD: Metabolic dysfunction-associated steatotic liver disease
MASH: Metabolic dysfunction-associated steatohepatitis
ME/CFS: Myalgic encephalomyelitis/chronic fatigue syndrome
miR: MicroRNA
mTOR: Mechanistic target of rapamycin
mTORC1: Mechanistic target of rapamycin complex 1
NF-κB: Nuclear factor kappa-B
NLRP3: NOD-, LRR- and pyrin domain-containing protein 3
PASC: Post-acute sequelae of SARS-CoV-2 infection
PCSK9: Proprotein convertase subtilisin/kexin type 9
PD: Parkinson disease
PI3K: Phosphoinositide 3-kinase
PINK1: PTEN-induced kinase 1
PLIN2: Perilipin-2
ROS: Reactive oxygen species
SARS-CoV-2: Severe acute respiratory syndrome coronavirus 2
siRNA: Small interfering RNA
SIRT1: Sirtuin-1
SQSTM1/p62: Sequestosome-1
TFEB: Transcription factor EB
ULK1: Unc-51-like autophagy activating kinase 1
VaD: Vascular dementia
VLDL: Very-low-density lipoprotein
VPS34: Vacuolar protein sorting 34
WAT: White adipose tissue
